# *p*-Hydroxylcinnamaldehyde induces the differentiation of oesophageal carcinoma cells via the cAMP-RhoA-MAPK signalling pathway

**DOI:** 10.1038/srep31315

**Published:** 2016-08-09

**Authors:** Ming Ma, Lian-mei Zhao, Xing-xiao Yang, Ya-nan Shan, Wen-xuan Cui, Liang Chen, Bao-en Shan

**Affiliations:** 1 Clinical Laboratory, The Fourth Hospital of Hebei Medical University, Shijiazhuang, Hebei 050011, P. R. China; 2 Research Center, The Fourth Hospital of Hebei Medical University, Shijiazhuang, Hebei 050011, P. R. China; 3 Department of Infection Management, The Fourth Hospital of Hebei Medical University, Shijiazhuang, Hebei 050011, P. R. China

## Abstract

p-Hydroxylcinnamaldehyde (CMSP) has been identified as an inhibitor of the growth of various cancer cells. However, its function in oesophageal squamous cell carcinoma (ESCC) and the underlying mechanism remain unclear. The aim of the present study was to characterize the differentiation effects of CMSP, as well as its mechanism in the differentiation of ESCC Kyse30 and TE-13 cells. The function of CMSP in the viability, colony formation, migration and invasion of Kyse30 and TE-13 cells was determined by MTS, colony-formation, wound healing and transwell assays. Western blotting and pull-down assays were used to investigate the effect of CMSP on the expression level of malignant markers of ESCC, as well as the activity of MAPKs, RhoA and GTP-RhoA in Kyse30 and TE-13 cells. We found that CMSP could inhibit proliferation and migration and induce Kyse30 and TE-13 cell differentiation, characterized by dendrite-like outgrowth, decreased expression of tumour-associated antigens, as well as the decreased expression of malignant markers. Furthermore, increased cAMP, p-P38 and decreased activities of ERK, JNK and GTP-RhoA, were detected after treatment with CMSP. These results indicated that CMSP induced the differentiation of Kyse30 and TE-13 cells through mediating the cAMP-RhoA-MAPK axis, which might provide new potential strategies for ESCC treatment.

Oesophageal carcinoma (EC) is the deadliest form of gastrointestinal malignancies, with a high incidence of approximately 0.4779 million new malignancies in China each year^1^. The most prevalent histologic type of EC is esophageal squamous cell carcinoma (ESCC)^2^. Although surgical intervention, radiotherapy and chemotherapy remain the treatments of choice for ESCC, unfortunately, the general death rate of ESCC patients remains greater than 60%, owing to recurrence, metastasis, advanced disease, and tumour multidrug resistance (MDR)^2^,^3^. Because of the markedly poor prognosis, there is an urgent need to identify novel and more effective strategies for ESCC treatment. Recently, studies concerning tumour cell differentiation have provided useful information for cancer treatment. Some agents have been reported to induce tumour cells including oesophageal cancer differentiation, such as all transretinoic acid (ATRA), a routine differentiation inducer in the treatment of AML-M3 leukaemia, 12-o-tetradecanoylphorbol-13-acetate (TPA) or forskolin^3^,^4^,^5^,^6^. However, drugs that function as oesophageal cancer differentiation inducers, especially chemical compounds extracted from traditional herbs, are extremely less developed.

Cochinchinamomordica seed (CMS) is the dried ripe seed of *Momordica cochinchinensis* (Lour.) Spreng. (Fam. Cucurbitaceae), and it has been traditionally used as a remedy to treat external carbuncle. It has been shown that CMS has potential effects on the immune response or as an adjuvant of immunity^7^. In addition to those effects, CMS has been widely used to treat various tumours in China, although its mechanisms have not yet been clearly elucidated^8^. *p*-Hydroxylcinnamaldehyde (CMSP) is a novel member of the ethanol extracts of CMS (CMSE). As we have shown previously, CMSP inhibited proliferation via inducing melanoma B16 F1 cell differentiation, and the MAPK signalling pathway may account partly for this function^2^,^8^; therefore, various components of this signalling pathway may serve as rational targets for the development of anticancer drugs^8^. This ability of CMSP to inhibit proliferation and induce differentiation in melanoma cells led to our hypothesis that it might also play a similar role in ESCC cells.

This study reports, for the first time, that CMSP can induce differentiation in ESCC cells, and the underlying mechanism of the anti-cancer effect of the CMSP on ESCC cell lines was investigated. These research findings will provide scientific support for the novel use of CMSP as an extract from traditional Chinese medicine for tumour diseases.

## Materials and Methods

### Ethics statement

All experimental methods were carried out in accordance with the approved guidelines. All experimental protocols were approved by Research Center, The Fourth Hospital of Hebei Medical University, Shijiazhuang, China.

### Materials

RPMI-1640, fetal bovine serum (FBS) and PBS were obtained from Gibco-BRL (Life technologies, Paisley, Scotland). ATRA is available from Sigma (Sigma Chemical Co., St Louis, MO, USA). Antibodies to total p44/42MAPK (ERK1/2), p-p44/42 (ERK1/2), total JNK/SAPK, p-JNK/SAPK, p38MAPK, p-p38MAPK and RhoA were all purchased from Cell Signaling Technology, Inc. (CST, CA, USA); Antibodies to C-myc, N-myc and β-actin were purchased from Abcam, Inc. (Cambridge, MA, USA); Antibodies against MMP2, E-cadherin, N-cadherin, and vimentin were supplied by Santa Cruz Biotechnology, Inc. (Santa Cruz, CA, USA); ELISA kits of cAMP, CEA and SCC were all purchased from Enzo Life Sciences Inc. (Farmingdale, NY, USA) and Nanjing Senbeijia Biological Technlogy Co., Ltd. (Nanjing, Jiangsu, China). ELISA kits of IL-6 and MIC-1 were all purchased from eBiosciences, Inc. (SanDiego, CA, USA) and MultiSciences Biotech Co., Ltd. (Hangzhou, Zhejiang, China). TRIzol reagent was purchased from Invitrogen (Carlsbad, CA, USA). Go Taq^®^ qPCR Master Mix was purchased from Promega (Madison, WI, USA). RevertAid™ First Strand cDNA Synthesis Kits was purchaseed from MBI Fermentas (Hanover, MD, USA). Annexin V-fluorescein isothiocyanate (FITC) and PI double stain was purchased from BD Pharmingen (San Diego, CA, USA). RhoA activation kit for pull-down assay was purchased from Neweast Biosciences (King of Prussia, PA, USA). The specific cAMP-inhibitor (SQ22536) was supplied by Sigma (Sigma Chemical Co., St. Louis, MO, USA) and dissolved in DMSO (final maximal concentration of dimethylsulfoxide (DMSO) in medium was 0.1% [v/v]) to yield a 1 mM stock solution.

### Cell lines

The human normal oesophageal cell line HEEC and human ESCC cell line Eca109 were obtained from the Cellular Biology Institute of the Shanghai Academy of Sciences (Shanghai, China). Kyse30 and Kyse180 cell lines were contibuted by professor Qimin Zhan (Chinese Peking Union Medical College, Beijing, China). TE-13 cell line was contibuted by professor Leiming Ren (Hebei medical university, Shijiazhuang, China). Both of these cell lines were cultured in RPMI-1640 medium containing 5% FBS, 100 U/ml penicillin and 100 μg/ml phytomycin at 37 °C and 5% CO_2_ in a humidified atmosphere incubator.

### Extraction and isolation of CMSP from CMS

Cochinchinamomordica seeds (CMS) were purchased from Lerentang Pharmacy of Shijiazhuang (Hebei Province, China) and were identified by Professor Ren (New Drug Research and Development Center of North China Pharmaceutical Group Corporation, Shijiazhuang, China). The dried CMS powder (10 kg) was extracted with 95% ethanol (2 × 30 L) under reflux for 2 h. The combined 95% ethanol extracts were concentrated under vacuum to produce a viscous residue. The residue was suspended in water (3 L) and then was extracted with P.E. (3 × 2 L) and EtOAc (3 × 2 L). The ethylacetate extracts were subjected to column chromatography on a PS25-300 column with a successive elution system of 60% acetone, 70% acetone, and 80% acetone. The 70% acetone portion (6.4 g) was subjected to silica gel column chromatography and eluted with chloroform, chloroform-acetone 5:1, 3:1 and 1:1, respectively. The chloroform fraction was applied to a Pre-HPLC column (15% CH_3_CN-H_2_O, Phenomenex 250 × 21.2 mm, 10 μm) to produce the *p*-hydroxylcinnamaldehyde compound, which was elucidated based on ESI-MS (ZMD Micromass, Micromass, England) and NMR spectrometry (INVOA500; Varian, USA) and termed CMSP in this study. CMSP was dissolved in 95% ethanol at a dose of 10 mg/ml for storage. The indicated concentration of CMSP in experiments was obtained by resolving it in serum-free RPMI-1640 medium (*in vitro*) or PBS (*in vivo*) before use, and the final concentration of ethanol was less than 0.5%.

### Cell viability assay

The effect of CMSP on ESCC cell viability was determined by the MTS (3-(4,5-dimethylthiazol-2-yl)-5-(3–carboxymethoxyphenyl)-2-(4- sulphophenyl)-2H-tetazolium) assay according to the manufacturer’s instructions (Promega, Madison, WI). The cells were plated at 1** **×** **10^4^ per well in 200 μl of RPMI1640 medium supplemented with 10% FBS and treated with CMSP (0, 10, 20 or 40 μg/ml) in 96-well plates (Gibco, USA). After incubation for 24, 48 or 72 h at 37 °C in a humidified incubator, MTS solution was added (20 μl/well) to the cells, which were incubated again for 2 h at 37 °C. The absorbance at 492 nm was measured using a microplate reader (TitertekMultiskan, North Ryde, Australia) to assess the effect of CMSP on cell viability.

### Cell morphology and differentiation

Briefly, 5** **×** **10^4^ Kyse30 and TE-13 cells were cultured in 6-well plates to evaluate the effect of CMSP on cell differentiation. After treatment with different doses of CMSP (0, 10, 20 or 40 μg/ml) or ATRA(10^−5^ mM) for 72 h, the cells were fixed with 4% paraformaldehyde for 15 min at room temperature and then were stained with Wright-Giemsa for 30 min. The cell differentiation rate was obtained as the number of cells with cytoplasmic extensions longer than three cellular bodies to the total number of cells.

### SEM morphology of Kyse30 and TE-13 cells treated with CMSP

The morphologic changes in the Kyse30 and TE-13 cells were observed by scanning electronic microscopy (SEM). Briefly, exponentially growing Kyse30 and TE-13 cells were seeded in 6-well plates at 10^6^ cells/well and were exposed to CMSP (20 μg/ml) or ATRA (10^−5^ mM) for 48 h. The cells were fixed in 2.5% glutaraldehyde and were washed three times with 0.075 M phosphate buffer (pH 7.4–7.6). The cells were fixed in 0.25% glutaraldehyde and dehydrated with increasing concentrations of ethanol (30, 50, 70, 80, 90, and 100%) and then embedded on coverslips. Cells on coverslips were dried in a vacuum dryer and coated with gold. Finally, the morphology of the cells was viewed using the S-3500n SEM system (Hitachi, Japan) at the Electron Microscopy Unit of Hebei Medical University (Shijiazhuang, China).

### RNA preparation and reverse transcription-quantitative polymerase chain reaction (RT-qPCR) analysis

The transcription of carcino-embryonic antigen (CEA), squamous cell carcinoma antigen (SCC), interleukin6 (IL-6) and macropphage inhibitory factor 1 (MIC-1) genes were detected by RT-qPCR analysis. Total RNA was extracted from Kyse30 and TE-13 cells using TRIzol reagent (Invitrogen, Shanghai, China) according to the manufacturer’s directions. The RNA concentration was routinely measured spectrophotometrically, and its quality was evaluated by visualization after agarose gel electrophoresis and ethidium bromide staining. First-strand cDNAs were generated from one-microgram aliquots of total RNA using RevertAid™ First Strand cDNA Synthesis Kits (Thermo) and incubation at 42 °C for 60 min. The resultant cDNAs were amplified by RT-qPCR using Go Taq^®^ qPCR Master Mix and specific primers for CEA, SCC, IL-6, MIC-1 and β-actin (Shanghai Generay Biotech; Supplementary Table 1), with the latter used as an internal control. PCR products were separated on 2% agarose gels and visualized by ethidium bromide staining. The relative level of expression of each target gene was assessed by the 2^−ΔΔCt^ method, where ΔΔCt = (Ct target gene of experimental group − Ct β-actin of experimental group) − (Ct target gene of control group − Ct β-actin of control group).

### Elisa

To investigate the content of cAMP, CEA, SCC, IL-6 and MIC-1 in the supernatants of Kyse30 and TE-13 cells or serum of balb-c/null mice treated with CMSP, ELISA was performed, according to the manufacturer’s instructions. Briefly, 2 × 10^5^ cells treated with 0, 10, 20 or 40 μg/ml CMSP were seeded in 6-well plates. The plates were incubated in a 5% CO_2_ humidified incubator at 37 °C, and the supernatants were collected after 0, 30, 60 and 120 min to detect the concentration of cAMP and after 24, 48 and 72 h to detect the concentrations of CEA, SCC, IL-6 and MIC-1.

### Western blot analysis

Kyse30 and TE-13 cells were lysed with 250 μl of lysis buffer (1% Triton X-100, 150 mM NaCl, 10 mM Tris-HCl, pH 7.4, 1 mM EDTA, 1 mM EGTA, pH 8.0, 0.2 mM Na_3_VO_4_, 0.2 mM phenylmethylsulphonylfluoride, and 0.5% NP-40). The lysates (40 μg per well of the SDS-PAGE gels) were subjected to sodium dodecyl sulphate polyacrylamide gel electrophoresis (SDS-PAGE) and were electrotransferred onto a polyvinylidene difluoride membrane (Millipore, Billerica, MA, USA). The membranes were incubated in PBS containing 5% bovine serum albumin for 2 h at room temperature, followed by overnight incubation at 4 °C with different dilutions of primary antibodies, including antibodies against C-myc (1:1000), N-myc (1:1000), P38 (1:2000), p-P38 (1:2000), ERK1/2 (1:2000), p-ERK1/2 (1:2000), SAPK/JNK (1:2000), p-SAPK/JNK (1:2000), RhoA (1:2000) and β-actin Ab (1:5000). The membranes were developed using the Odyssey infrared imaging system (LI-COR, USA). according to the manufacturer’s instructions. The levels of protein in each sample were normalized relative to those of β-actin. Each experiment contained triplicate wells of each sample, and all of the experiments were repeated at least three times.

### Flow cytometry assay

The effect of CMSP on apoptosis was assessed by flow cytometry analysis of cells incubated with Annexin V-fluorescein isothiocyanate (FITC) and PI double staining according to the manufacturer’s instructions. Cell cycle analysis was performed using PI staining. Single-cell suspensions of Kyse30 and TE-13 cells (1 × 10^6^ cells per sample) were fixed in 70% ice-cold ethanol and treated with RNase A. After staining with PI for 15 min, the cells were resuspended in ice-cold PBS and were analysed using a fluorescence activated cell sorting (FACS) flow cytometer (FACSCalibur™; Becton-Dickinson, USA). The data were analysed using CellQuest Pro software and were expressed as the mean ± SD of the mean of at least three independent experiments.

### Colony formation assay

To investigate the effects of CMSP on the colony formation of ESCC cells, the colony forming assay was performed. Briefly, 500 Kyse30 or TE-13 cells were seeded on 6-well plates and cultured in a humidified incubator with 5% CO_2_ for 10 days. RPMI1640 medium supplemented with 10% FBS was changed every 72 hours. Ten days later, the cell colonies were fixed with 4% paraformaldehyde, stained with crystal violet (2%) for 10 minutes and counted.

### Tumour cell migration and invasion assays

The tumour cell migration assay was performed in a 24-well transwell chamber (Collaborative Biomedical, Becton Dickinson Labware, Bedford, MA), which contained an 8-μm pore size polycarbonate membrane filter and was precoated with 100 μg of Matrigel for invasion assays (Becton-Dickinson, Bedford, USA). Briefly, 2 × 10^5^ cells were seeded in the upper chambers and incubated in 500 μl RPMI medium (without FBS) with 0, 10, 20 or 40 μg/ml CMSP, while 500 μl medium with 10% FBS was placed in the lower chambers. The plates were incubated for 24 h in a 5% CO_2_ humidified incubator at 37 °C. Cells on the upper side of the filters were removed using cotton- tipped swabs, and the filters were washed with PBS. Next, the cells on the lower side were fixed in 4% formaldehyde and stained with 1% crystal violet in PBS for 5 min at room temperature. The cells on the lower side of the filters were defined as migration cells and were counted at ×200 magnification in 5 random fields of each filter.

Tumour cell migration was also assessed using the wound healing assay. Kyse30 and TE-13 cells were seeded at 2 × 10^4^/well. After scraping the cell monolayer with a sterile micropipette tip, the wells were washed with serum-free medium three times. The first image of each scratch was acquired at time zero. After 48 h, each scratch was examined and captured at the same location, and the healed area was measured.

### GTP-RhoA pull-down assay

A pull-down assay was carried out to determine GTP-RhoA activity in the ESCC cell lines treated with 20 μg/ml CMSP after 0, 30, 60 and 120 min according to the manufacturer’s instructions. Briefly, the Kyse30 and TE-13 cells were homogenized in a lysis buffer and then were cleared by a 10-min centrifugation at 12,000 *g* at 4 °C. The supernatants were then incubated with the RhoA assay reagent. The RhoA binding beads were collected by centrifugation and then were washed three times with lysis buffer. The bead-binding complexes were then subjected to western blot analysis to determine the amount of GTP-RhoA.

### *In vivo* tumour growth assay

Balb-c/null mice were used in the *in vivo* tumour growth assay. Care was provided according to the National Research Council Guide for the Care and Use of Laboratory Animals and was approved by the Institutional Animal Care and Use Committee (IACUC) of Hebei Medical University, Shijiazhuang, China. Kyse30 cells were harvested with trypsin solution and resuspended in PBS. Cells (1 × 10^6^ cells/mouse) in 0.1 ml were injected subcutaneously into balb-c/null mice. The stock solution of CMSP, CDDP and ATRA was resolved in PBS, and the final concentration of ethanol was less than 0.5%. The mice were divided randomly to five groups (6 mice/group) and were injected paratumor since the 9^th^ day: GI: Control group treated with PBS once every two days ; GII: CMSP (10 mg/kg) group treated once every two days; GIII: CMSP (20 mg/kg) group treated once every two days; GIV: Cisplatin (CDDP) (2 mg/kg) group treated once every two days; and GV: ARTA (10^−2^ mmol/kg) group treated once every two days and all mice were sacrificed by cervical dislocation on day 32 after drawing blood, and the tumour, liver, spleen and lungs were removed, washed with PBS, and stained with haematoxylin and eosin (H&E). Immunohistochemical staining was performed to detect the expression of N-myc and C-myc in tumour tissues. The concentration of CEA, SCC, IL-6 and MIC-1 in serum of mice was detected using ELISA.

### Immunohistochemistry

Immunohistochemical analysis was performed according to a previous study using the streptavidin-peroxidase (SP) method^9^. After fixation with 10% formalin, the paraffin-embedded tumour tissues were cut into 4-μm-thick sections. The sections were dewaxed and rehydrated with xylene and ethanol. Endogenous peroxidase activity was blocked with 3% H_2_O_2_ in deionized water for 15 min. After blocking with 1% goat serum, the antigens of the sections were retrieved in a pressure kettle in Tris-EDTA buffer (pH 9.0) for 10 min and were cooled at room temperature. The sections were then incubated with primary antibodies against N-myc and C-myc for 3 h at 37 °C, followed by biotinylated secondary antibody and streptavidin-biotinylated horseradish peroxidase complex (Zhongshan, Beijing, China), after washing for three times (5 min every time). Protein expression was visualized and classified based on the percentage of positive cells and intensity of staining.

### Statistical analysis

The data are reported as the mean value ± SD. One-way analysis of variance (ANOVA) was performed to determine the significance between groups. Turkey’s method was used for multiple comparisons. P-values less than 0.05 were considered to indicate statistical significance. Data were obtained from at least three independent experiments with a similar pattern. All of the data analyses were performed using SPSS13.0.Software.

## Results

### Inhibition of ESCC cell proliferation by CMSP isolated from EtOAc extracts of CMS

**Figure 1:**
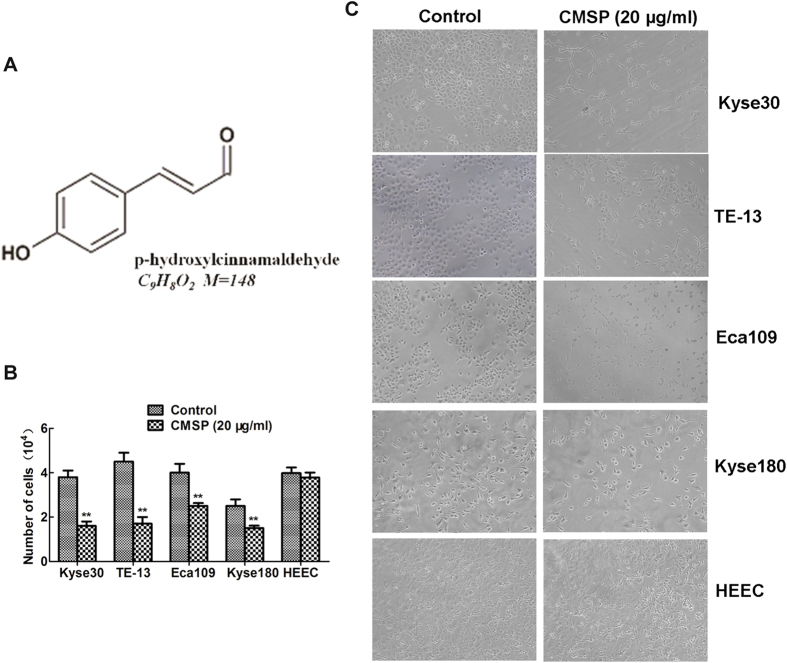
Chemical structure of CMSP and its inhibitory effect on ESCC cells. (**A**) Structure of CMSP. (**B**,**C**) The inhibition effects of CMSP isolated from CMS on the number and morphology of ESCC and normal oesophageal cells were determined. The data presented are means ± SD from at least three independent experiments (×100). **P < 0.01, compared with the control group.

First, we isolated CMSP from the pure chloroform-eluted fraction, and its structure was identified based on spectral analysis (MS, 1H NMR, 13C NMR; Supplementary Table 2 and Fig. 1A). Our previous report had shown that CMSP could inhibit the proliferation and induce differentiation of B16 F1 cells^8^. To further address whether CMSP can mediate the inhibition and differentiation ability of ESCC, the effect of CMSP on the inhibition of various ESCC cell lines, including Kyse30, TE-13, Eca109 and Kyse180, was evaluated. Our results showed that the growth of these cells was significantly inhibited by 20 μg/ml CMSP for 72 h (Fig. 1B,C). The numbers of these cells treated with CMSP were decreased significantly (Fig. 1B). By contrast, the growth and morphology of the normal oesophageal cell line HEEC were not changed (Fig. 1B). These results suggested that CMSP might predominantly target malignant cells but has no influence on normal cells. The concentrations of CMSP that could induce 50% cell proliferation inhibition of Kyse30, TE-13, Eca109 and Kyse180 cell lines were 15.13 ± 2.05 μg/ml, 17.27 ± 2.62 μg/ml, 26.47 ± 3.27 μg/ml and 25.40 ± 3.11 μg/ml, respectively, indicating that p-hydroxylcinnamaldehyde had a more marked inhibition effect on Kyse30 and TE-13 cells. Meanwhile, CMSP induced morphologic changes in Kyse30 and TE-13 cells analogous to B16 F1 cells treated with 20 μg/ml CMSP which has been verified to induce B16 F1 differentiation. Therefore, Kyse30 and TE-13 cells were used in subsequent experiments for elucidate the function and underlying mechanism of CMSP were explored in this study.

### Effects of CMSP on the viability and differentiation of Kyse30 and TE-13 cells

**Figure 2:**
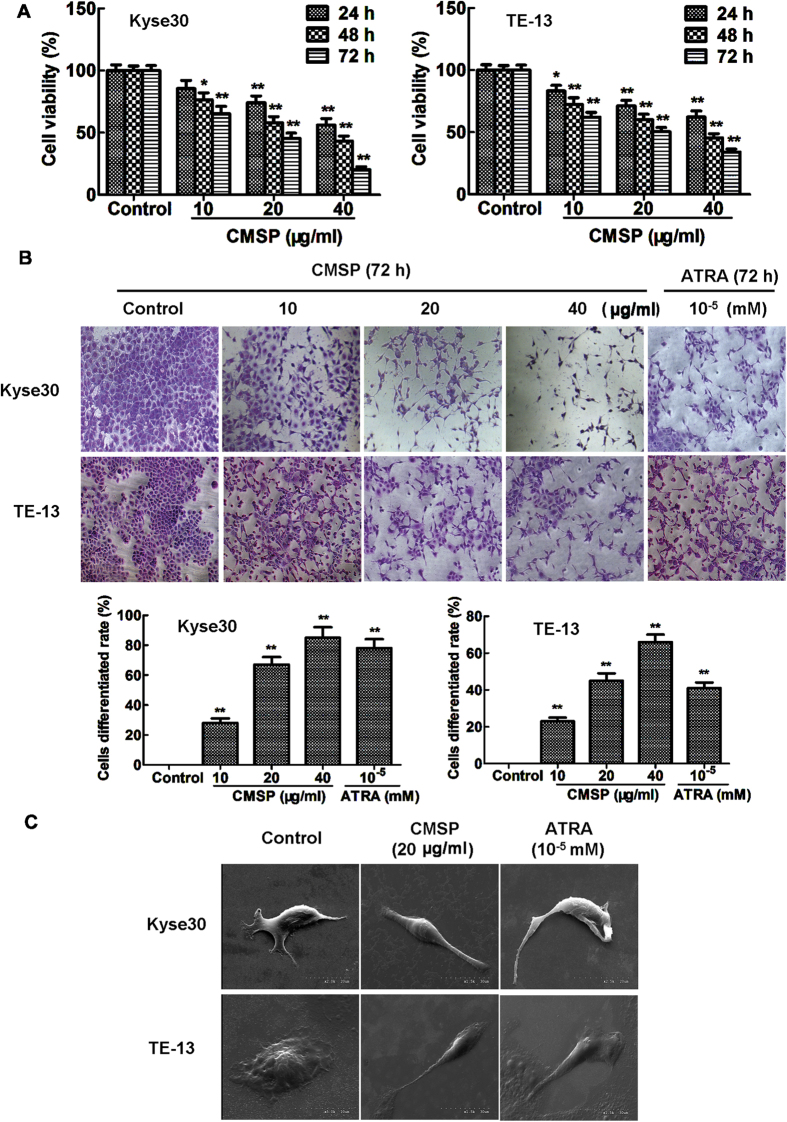
Effects of CMSP on the viability and morphology change in Kyse30 and TE-13 cells. (**A**) The effect of 10–40 μg/ml CMSP on the viability of Kyse30 and TE-13 cells was detected by the MTS assay. The data presented are means ± SD from at least three independent experiments. *P < 0.05, **P < 0.01, compared with the control group. (**B**) Morphology change and differentiated cell rate caused by CMSP or ATRA, as determined by Giemsa staining. Representative histograms as the percentage of dendrite-like cells in CMSP-treated Kyse30 and TE-13 cells (×100). The data presented are means ± SD from at least three independent experiments. **P < 0.01, compared with the control group. (**C**) The morphology change in Kyse30 and TE-13 cells was investigated by scanning electron microscopy.

To further evaluate the novel effect of CMSP on ESCC cell viability, Kyse30 and TE-13 cells were exposed to different concentrations of CMSP (10, 20 or 40 μg/ml) for 24, 48 and 72 h. As shown in Fig. 2A, the viability of Kyse30 and TE-13 cells was inhibited in a dose- and time-dependent manner. These data indicated that CMSP could effectively and significantly suppress the viability of Kyse30 and TE-13 cells.

To explore the function of CMSP in inducing the differentiation of oesophageal cancer cells, Wright-Giemsa staining was performed to observe the morphological changes in Kyse30 and TE-13 cells. As shown in Fig. 2B, 10–40 μg/ml CMSP-treated cells showed typical dendrite-like cellular protrusions, and the percentage of such elongated cells was significantly and progressively increased with the increase in CMSP concentration. The morphological changes in Kyse30 and TE-13 cells treated with CMSP were analogous to those induced by ATRA, which is a well-known agent that can induce differentiation in various tumour cells, including oesophageal cancer cells^3^. In addition, electron microscopy also exhibited that CMSP-treated Kyse30 and TE-13 cells showed a dendrite-like morphology (Fig. 2C).

**Figure 3:**
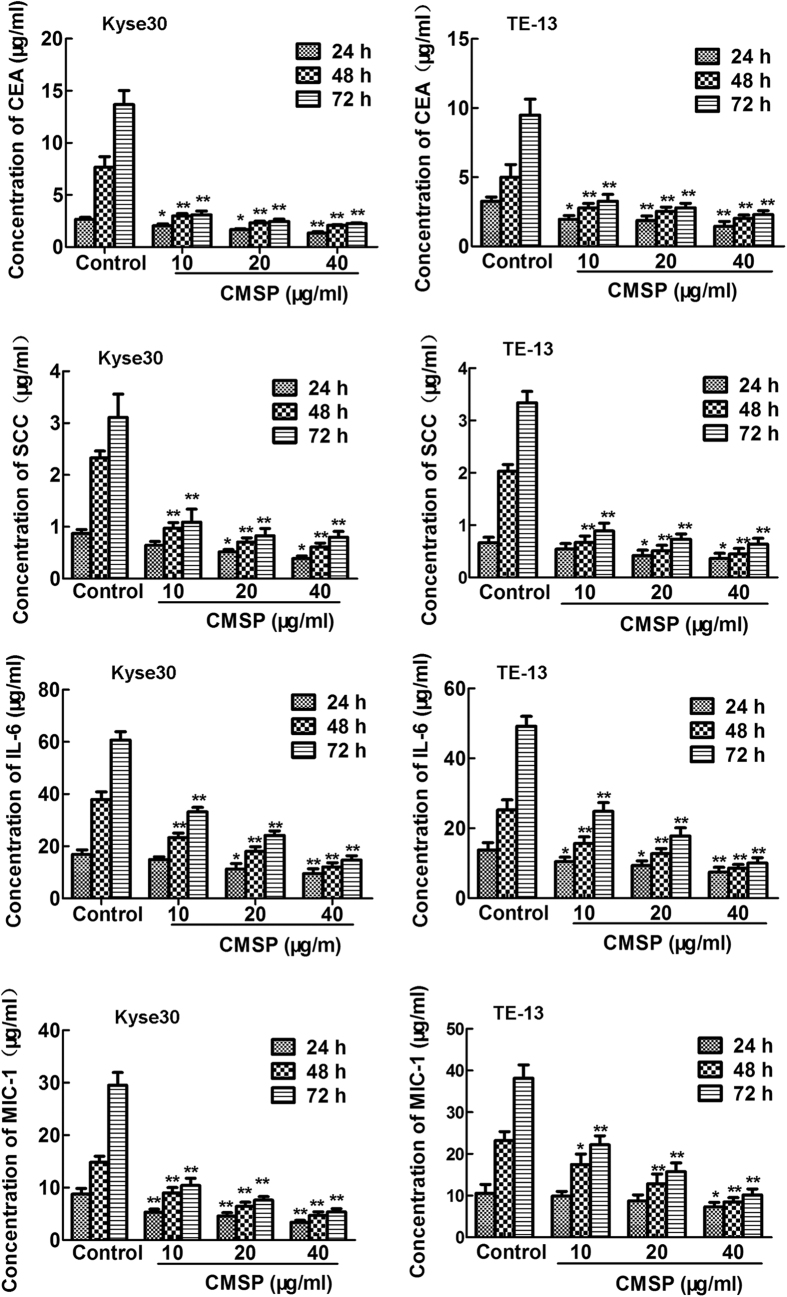
Effects of CMSP on tumour-related antigen secretion and malignant marker expression of Kyse30 and TE-13 cells caused by CMSP. The content of CEA, SCC, IL-6 and MIC-1 in the supernatants of Kyse30 and TE-13 cells was determined using ELISA. The data presented are means ± SD from at least three independent experiments. *P < 0.05, **P < 0.01, compared with the control group.

It is well known that expression of typical tumour related antigens (TAA) CEA and SCC is negtive parameter of ESCC differentiation and prognosis^9^,^10^,^11^. Moreover, interleukin-6 (IL-6) and macropphage inhibitory factor 1 (MIC-1), the two important cytokines expressed in various cancer cells, are extensively regarded as a malignant biomarker of ESCC^12^,^13^,^14^,^15^. The effect of CMSP on the expression of CEA, SCC, IL-6 and MIC-1 in mRNA in Kyse30 and TE-13 treated with CMSP was measured to further verify the effect of CMSP. Furthermore, we evaluated the CEA, SCC, IL-6 and MIC-1 content in the supernatants of Kyse30 and TE-13 cells treated with CMSP. As shown in Fig. 3 and Supplementary Fig. 1, CMSP could decrease the expression of CEA, SCC, IL-6 and MIC-1 both in mRNA and protein secretion levels significantly in a dose- and time- dependent manner, compared with the control group.

The overexpression of C-myc and N-myc is also widely considered to be a malignant biomarker of various cancer cells^16^,^17^,^18^. Western blot analysis showed that C-myc and N-myc proteins were all decreased significantly after treatment with CMSP in a dose- and time- dependent manner, suggesting that the malignant phenotype of Kyse30 and TE-13 cells was mitigated by CMSP (Supplementary Fig. 2).

In summary, these results indicated that CMSP can induce Kyse30 and TE-13 differentiation.

### The cell cycle is arrested in G0/G1 phase and apoptosis is not involved in the growth inhibition of Kyse30 and TE-13 cells by CMSP

The results of flow cytometry revealed that CMSP increased the number of cells in G0/G1 phase and decreased the number of cells in S phase in Kyse30 and TE-13 cells, showing that CMSP significantly prevented cell cycle exit from G1 phase arrest (Supplementary Fig. 3).

Flow cytometry was also performed to estimate the rate of apoptosis by quantitative assessment of Annexin V/PI-stained Kyse30 and TE-13 cells. As shown in Supplementary Fig. 4, CMSP treatment did not significantly increase the number of Annexin V-FITC-positive cells compared with that in the control group. Thus, apoptosis may not be involved in the growth arrest of Kyse30 and TE-13 cells treated with CMSP.

### Inhibition of colony formation and metastasis of Kyse30 and TE-13 cells by CMSP

**Figure 4:**
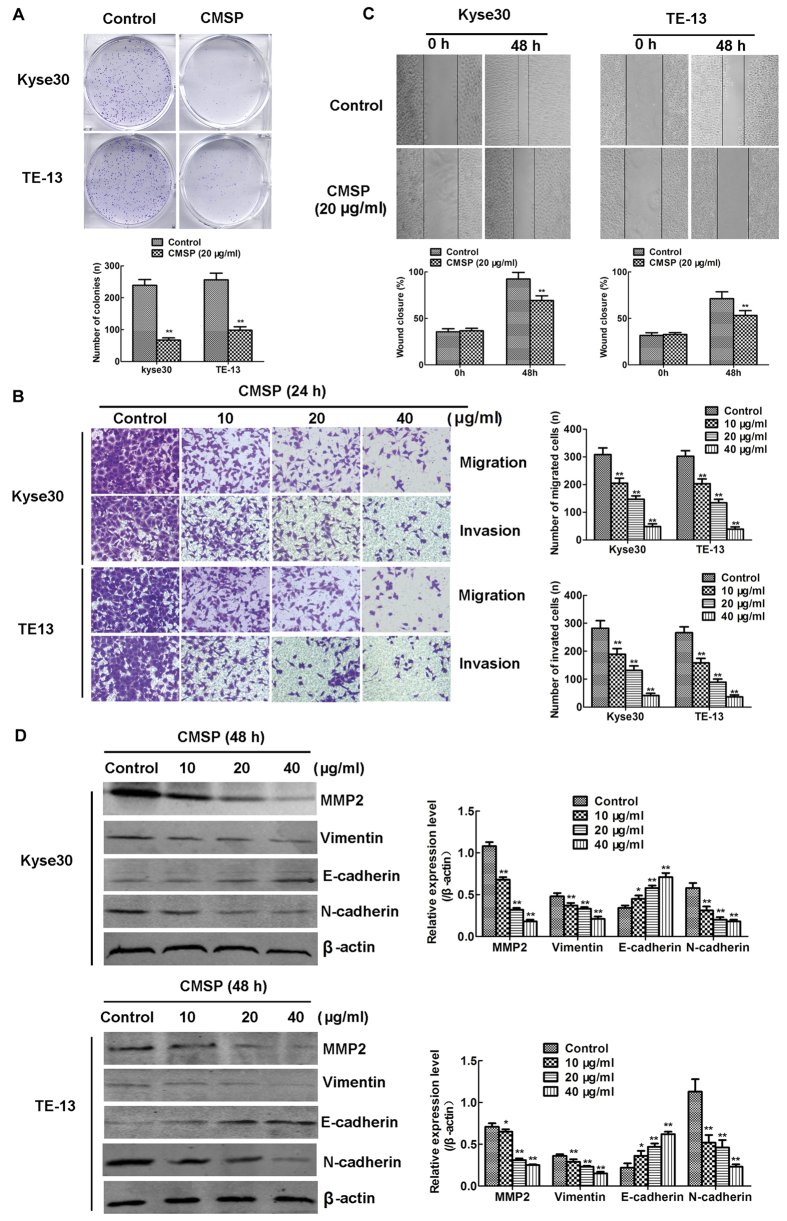
Effects of CMSP on the colony formation, migration and invasion of Kyse30 and TE-13 cells. (**A**) Significant inhibition of cell colony formation was observed upon treatment with CMSP (20 μg/ml) for 10 days. The number of colonies was calculated and plotted on the histogram (n = 3). **P < 0.01, compared with the control group. (**B**) Effect of CMSP on the migration and invasion ability following exposure to different concentrations (10, 20 or 40 μg/ml) for 24 h was investigated by the transwell assay. The number of migrated and invaded cells was calculated (n = 3). **P < 0.01, compared with the control group. (**C**) Effect of CMSP on the mobility ability of Kyse30 and TE-13 cells after exposure for 48 h was investigated by the wound healing assay. The wounds were photographed, and the wound closure percentage from a representative experiment (n = 3) was temporally measured using Axio Vison software. **P < 0.01, compared with the control group. (**D**) The protein levels of the EMT-related proteins MMP2, E-cadherin N-cadherin, and vimentin, as measured by western blotting. β-Actin served as a loading control. The data presented are means ± SD from at least three independent experiments. *P < 0.05, **P < 0.01, compared with the control group.

Colony formation and metastasis are two main malignant features of ESCC cells. The numbers and volume of colonies of Kyse30 and TE-13 cells *in vitro* were reduced after treatment with CMSP (20 μg/ml) (Fig. 4A). The migration assay showed that the numbers of cells migrating into transwell filters after treatment with CMSP at concentrations of 10, 20 and 40 μg/ml were 308, 205, 147 and 47 of Kyse30, and 302, 203, 134 and 39 of TE-13, respectively (Fig. 4B), which were consistent with the results that the numbers of invading cells after treatment with 10, 20 of 40 μg/ml CMSP (282, 189, 131 and 41 of Kyse30, and 266, 158, 89, and 36 of TE-13 respectively) (Fig. 4B). Wound healing experiments also revealed that the migration of Kyse30 and TE-13 cells was inhibited after treatment with 20 μg/ml CMSP for 48 h (Fig. 4C).

The epithelial-to-mesenchymal transition (EMT) is considered an important part of the mechanism of tumour metastasis^19^,^20^,^21^. Intrinsically, there are several common molecular markers for the EMT of ESCC, including decreased expression of the epithelial marker protein E-cadherin and increased expression of the mesenchymal markers N-cadherin and vimentin^19^,^20^,^21^. Additionally, it is well known that the matrix metalloproteinase (MMP) family is essential for the metastasis of oesophageal cancer cells^19^. As shown in Fig. 4D, the levels of the expression of N-cadherin, vimentin and MMP2 proteins were reduced, whereas the level of the epithelial marker protein E-cadherin was increased, in CMSP-treated Kyse30 and TE-13 cells, compared with the control. Thus, our results revealed that CMSP can inhibit ESCC metastasis through blocking EMT.

Taken together, these results indicated that CMSP could inhibit Kyse30 and the TE-13 cell malignant phenotype.

### Influence on the cAMP-RhoA-MAPK signalling pathway may be involved in CMSP-induced differentiation of Kyse30 and TE-13 cells

**Figure 5:**
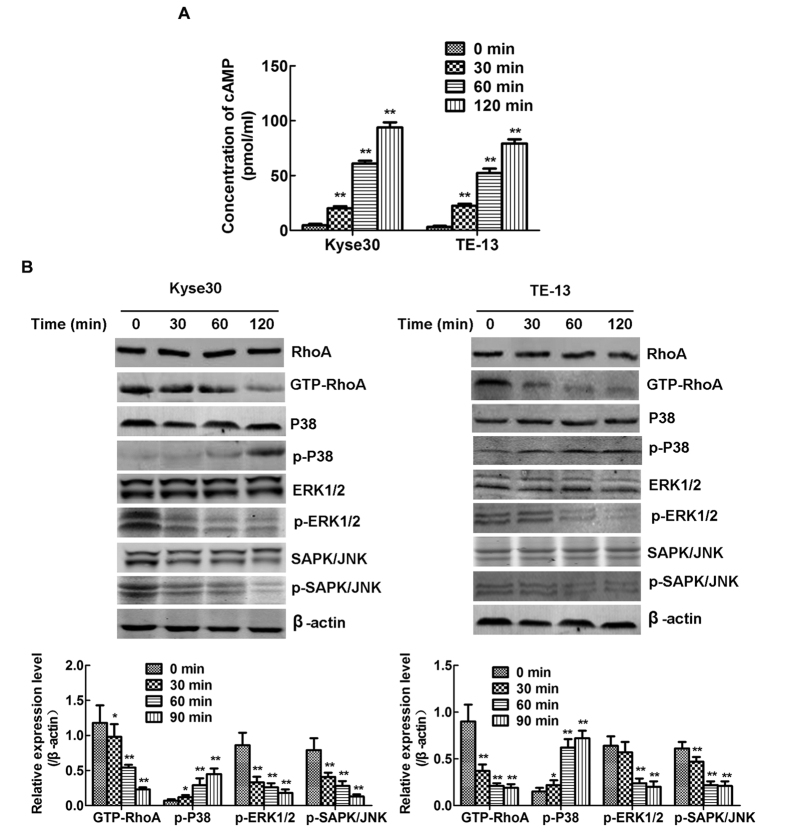
Effects of CMSP on cAMP production, RhoA and GTP-RhoA expression and the phosphorylation of MAPKs in Kyse30 and TE-13 cells. (**A**) The content of cAMP in the supernatants of Kyse30 and TE-13 cells treated with CMSP was assayed by ELISA. The data presented are means ± SD from at least three independent experiments. *P < 0.05, **P < 0.01, compared with the control group. (**A**) Kyse30 and TE-13 cells were treated with 20 μg/ml CMSP for 30, 60 and 120 min. The GTP-RhoA, phosphorylated ERK, p38, and JNK kinase proteins in the cell lysate were assayed by western blotting. β-Actin was used as an internal control. The data presented are means ± SD from at least three independent experiments. *P < 0.05, **P < 0.01, compared with the control group.

To determine the signalling pathway that is influenced by CMSP, the effect of CMSP on the activity of MAPK signalling, which is involved in Kyse30 and TE-13 cell differentiation, was evaluated. As shown in Fig. 5B, CMSP significantly suppressed the activities of the ERK and JNK signalling pathways while enhancing the P38 signalling pathway. Western blot assay also showed that GTP-RhoA was downregulated by CMSP (Fig. 5B). It is well known that the GTP-RhoA signalling pathway functions upstream of the MAPK signalling pathway and is regulated by cAMP^22^,^23^. In previous studies regarding the expression of GTP-RhoA, the activation status of RhoA was shown to be antagonized by the phosphorylation of Ser188 by PKA, the main effector of cAMP^21^,^22^,^23^. ELISA also revealed that the cAMP content was significantly increased in the culture supernatant of Kyse30 and TE-13 cells treated with CMSP (Fig. 5A). Thus, increased cAMP induced by CMSP might be an initial factor regulating the differentiation of Kyse30 and TE-13 cells.

Furthermore, to elucidate the underlying mechanism of signalling pathway in CMSP-induced cells differentiation, the effect of specific cAMP inhibitor (SQ22536) affecting on growth and differentiation of Kyse30 and TE-13 were investigated. Kyse30 and TE-13 cells were pre-treated with SQ22536 (20 μM) for 1 h, followed by treatment with or without 20 μg/ml CMSP. The results were presented in Supplementary Fig. 5A,B, showing that SQ22536 had no significant effect on proliferation and cycle distribution of Kyse30 and TE-13 cells without CMSP; however, SQ22536 blocked cells proliferative inhibition and G0/G1 phase arrest in Kyse30 and TE-13 cells treated with 20 μg/ml CMSP. SQ22536 aslo impeded the morphologic changes induced by CMSP (Supplementary Fig. 6A). Moreover, block of cAMP by SQ22536 markedly increased the expression of CEA, SCC, IL-6 and MIC-1 in Kyse30 and TE-13 cells treated with 20 μg/ml CMSP in protein secretion level (Supplementary Fig. 6B). The expression of C-myc and N-myc was aslo upregulated significantly by SQ22536 in Kyse30 and TE-13 cells treated with CMSP (Supplementary Fig. 6C). Furthermore, ELISA revealed that the cAMP content was significantly decreased by SQ22536 in the culture supernatant of Kyse30 and TE-13 cells treated with CMSP (Supplementary Fig. 7A). And the expression of GTP-RhoA, p-ERK1/2 and p-SAPK/JNK were upregulated significantly by SQ22536, while the expression of p-P38 was not changed in Kyse30 and TE-13 cells treated with CMSP (Supplementary Fig. 7B).

Taken together, the above data indicated that there is an axis of cAMP- GTP-RhoA-ERK/JNK regulating the differentiation of Kyse30 and TE-13 cells, which could be affected by CMSP. These results were in good agreement with our previous reports on the CMSP-induced differentiation of B16 F1 cells^8^; however, the mechanism underlying the activation of the P38 pathway remains unknown.

### Effect of CMSP on the growth and differentiation of Kyse30 cells *in vivo*

**Figure 6:**
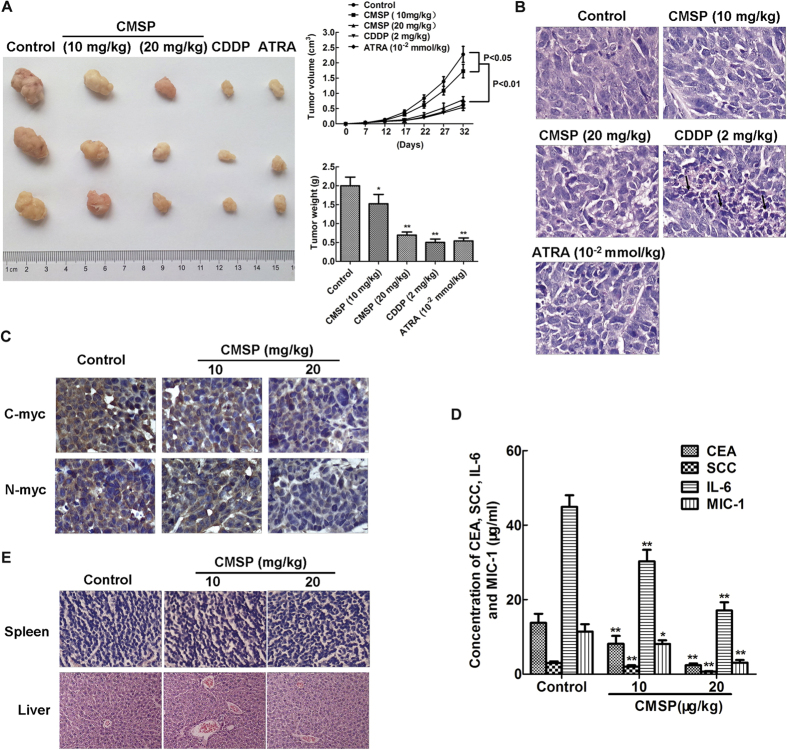
Inhibition of Kyse30 cell growth *in vivo* by treatment with CMSP. (**A**) CMSP treatment inhibited tumour growth of Kyse30 cells *in vivo*. Left: Representative images of tumours formed in nude mice. Right: Tumour growth curves of xenograft tumours and measurement of the tumour weight (n = 6 for each group). (**B**) Histological analysis of the tumour tissues of mice in all of the groups. Representative H&E images are shown (×400). (**C**) Representative immunohistochemical images showing the expression levels of C-myc and N-myc in the tumour tissues of both groups (×400). (**D**) The content of CEA, SCC, IL-6 and MIC-1 in the serum of nude mice treated with CMSP was assayed by ELISA. The data presented are means ± SD from at least three independent experiments. **P < 0.01, compared with the control group. (**E**) Histological analysis of sections from the spleen and livers of mice treated with CMSP or untreated mice. Representative H&E images are shown (×400).

In this study, the ESCC model was used to evaluate the effect of CMSP on Kyse30 cell growth in mice. The volume and weight of tumours in mice treated with CMSP, ATRA or CDDP at different doses were significantly lower than those in control mice (Fig. 6A). Histology showed that no apoptotic and necrotic cancer cells were found in control, CMSP-treated and ARTA-treated tumours; by contrast, conspicuous apoptotic and necrotic cancer cells were seen in CDDP-treated tumours (Fig. 6B). Immunohistochemistry showed that CMSP-treated tumours expressed lower levels of the malignant markers C-myc and N-myc (Fig. 6C). ELISA also revealed that the concentration of CEA, SCC, IL-6 and MIC-1 in the serum of CMSP-treated mice were lower than those in the serum of control mice (Fig. 6D). Moreover, no pathological changes were noticed in the liver and spleen tissues in both the control and CMSP-treated groups (Fig. 6E). All of the above indicated that the differentiation of Kyse30 cells may be induced *in vivo*.

## Discussion

A potentially less toxic approach to treat cancer is using agents that can induce cancer cell differentiation, termed ‘differentiation therapy’, which has become a novel therapeutic approach aimed at modifying tumour cell proliferation to a slower rate and decreasing its earlier neoplastic attributes^8^. Our previous study has shown that CMSE could inhibit proliferation and induce the differentiation of B16 F1 cells *in vitro* and *in vivo*^8^. In this study, we isolated *p*-hydroxylcinnamaldehyde (CMSP) from CMSE, and it was very powerful in inducing ESCC cell differentiation, which was characterized by proliferation inhibition, cell cycle arrest, dendrite-like outgrowth, and decreased expression of tumour markers. Actually, several agents can induce oesophageal cancer cell differentiation, including cAMP-elevating agents, ATRA, TPA, and forskolin^3^,^4^,^5^. For the first time, this study demonstrated that CMSP, a Chinese herbal extract, could induce the differentiation and inhibit the proliferation and metastasis of ESCC cells.

First, we found that 10–40 μg/ml CMSP could effectively and significantly suppress the proliferation of Kyse30 and TE-13 cells in a dose- and time-dependent manner. The cell cycles of the two cell lines were arrested in G0/G1 phase, and no apoptotic cells were observed in Kyse30 and TE-13 cells treated with CMSP. These experimental findings suggested that inducing apoptosis may not be involved in the growth inhibition of Kyse30 and TE-13 cells by CMSP.

The morphological correlates of differentiation have been known to pathologists for over one hundred years^8^. In this study, Kyse30 and TE-13 cells grew dendrite-like outgrowths after treatment with 10–40 μg/ml CMSP for 24, 48 or 72 h. The morphology change was also confirmed by a dendrite-like contour observed under the electron microscope.

It is well known that the typical tumour-related antigens (TAA) CEA and SCC express in a high level during early embryonic development of squamous epithelium including esophagus, and gradually disappear in the process of mature differentiation. It has been reported that the expression level of CEA and SCC was in a negatively relationship with the differentiation and prognosis of ESCC^9^,^10^,^11^. Additionally, IL-6 and MIC-1, the two of the most important cytokines widely existing in tumour microenvironment, play an important part in diverse biological events, including cell proliferation, differentiation, development, apoptosis, neoplastic change and immune escape^12^,^13^. High expression of IL-6 and MIC-1 is observed in ESCC tissue, and positively correlated with tumour progression, low differentiation and poor prognosis^13^,^14^,^15^. Thus the expression IL-6 and MIC-1 is extensively regarded as a malignant biomarker of ESCC. In the present study, decreased tumour-related antigen (CEA and SCC) and malignant biomarkers (IL-6 and MIC-1) expression at both mRNA and protein secretion levels in CMSP-treated cells indicated that oesophageal cancer cells lost the malignant phenotype gradually.

It has been reported that elevated C-myc and N-myc levels promote cell proliferation and neoplastic changes through oncogenic signalling within cells^16^,^17^. Furthermore, the overexpression of C-myc and N-myc is widely observed in various cancers, including oesophageal cancer, and accumulating data have indicated that the increased expression of C-myc and N-myc blocks differentiation^14^,^15^. In our study, C-myc and N-myc levels were both decreased significantly after treatment with CMSP, suggesting that the malignant phenotype of Kyse30 and TE-13 cells was mitigated by CMSP. Additionally, the colony formation, EMT, migration and invasion abilities of Kyse30 and TE-13 cells were all decreased after CMSP treatment, also confirming the functions of inducing differentiation.

To further investigate the molecular mechanism of CMSP-induced Kyse30 and TE-13 cell differentiation, we analysed the activities of the MAPK and RhoA signalling pathways. It is known that MAPKs, such as ERK 1/2, SAPK/JNK, P38 and ERK5/BMK, play important roles in the differentiation of various cancer cells, including ESCC^24^,^25^,^26^,^27^. In addition, the activation of P38 and inhibition of the ERK and SAPK/JNK pathways have been reported to stimulate differentiation in various tumour cells^8^,^27^. In our previous study, increased P38 and decreased activities of ERK1/2 and SAPK/JNK pathways were detected in melanoma B16 F1 cells treated with CMSP extract^2^,^8^. In this study, CMSP also resulted in similar results-i.e., the enhanced activity of P38 and decreased activities of ERK1/2 and SAPK/JNK pathways in Kyse30 and TE-13 cells. Moreover, GTP-RhoA is an upstream activator of the MAPK pathway, its expression is downregulated by cAMP, and it affects the differentiation of cancer cells^24^,^25^,^28^,^29^,^30^,^31^. In the present study, we found that decreased GTP-RhoA and increased cAMP in CMSP-treated cells, indicated that cAMP- induced RhoA activation blockade might be a key factor affected by CMSP regulating the differentiation of cancer cells. Furthermore, the hypothesis was confirmed by that blockade of cAMP by SQ22536 markedly upregulated expression of GTP-RhoA, p-ERK1/2 and p-SAPK/JNK and impeded CMSP-induced differentiation of the Kyse30 and TE-13 cells. However, the role of P38 pathway in CMSP-induced ESCC cell differentiation remains to be further studied.

The *in vivo* model showed that tumour growth was significantly slower in CMSP-treated mice. Lower levels of CEA, SCC, IL-6 and MIC-1 in the serum and C-myc and N-myc in tumours of CMSP-treated mice also indicated that xenograft cells exhibited a relatively benign phenotype. Moreover, no pathological changes were noticed in the liver and spleen tissues in both groups, indicating that there might be no toxicity and damage in mice treated with CMSP in liver and spleen tissue.

In conclusion, CMSP from CMSE has effects on the proliferation, metastasis and differentiation of ESCC cells. Thus, CMSP might be a novel and potential drug in clinical and preclinical applications to treat oesophageal carcinoma.

## Additional Information

**How to cite this article**: Ma, M. *et al*. *p*-Hydroxylcinnamaldehyde induces the differentiation of oesophageal carcinoma cells via the cAMP-RhoA-MAPK signalling pathway. *Sci. Rep*. **6**, 31315; doi: 10.1038/srep31315 (2016).

## Supplementary Material

Supplementary Information
