# Supplementary material for: p-Hydroxylcinnamaldehyde induces the differentiation of oesophageal carcinoma cells via the cAMP-RhoA-MAPK signalling pathway
Source: Sci Rep. 2016 Aug 9;6:31315. doi: 10.1038/srep31315 (PMC4977536; doi:10.1038/srep31315)
Supplement: Supplementary Information [file srep31315-s1.pdf]

p-Hydroxylcinnamaldehyde induces the differentiation of oesophageal carcinoma cells via the cAMP-RhoA-MAPK signalling pathway

Ming Ma [1,2#], Lian-mei Zhao [2#], Xing-xiao Yang [3], Ya-nan Shan [3], Wen-xuan Cui [2], Liang Chen [2] & Bao-en Shan [2*]

1 Clinical Laboratory, The Fourth Hospital of Hebei Medical University, Shijiazhuang, Hebei 050011, P. R. China;

2 Research Center, The Fourth Hospital of Hebei Medical University, Shijiazhuang, Hebei 050011, P. R. China.

3 Department of Infection Management, The Fourth Hospital of Hebei Medical University, Shijiazhuang, Hebei 050011, P. R. China;

[#]Ming Ma and Lian-mei Zhao contributed equally to this work

* **Correspondence to:** Baoen Shan Professor

E-mail: shanbaoen@163.com

Tel: 86311-86095283

Address: 12# Jiankang road, Shijiazhuang, China

Supplementary table 1. Primer sequences for reverse transcription-quantitative polymerase chain reaction

Gene	Primer sequence	Annealing temperature (°C)
β-actin	Forward 5′-GTTGTGATGGGTTCTGA-3′	60
	Reverse 5′-GAGCAATAGCGTCTGTG-3′	
CEA	Forward 5′-CGCTGAGTTCCTGCGTACC-3′	60
	Reverse 5′-TCTGCGGTGCTGTTGTGG-3′	
SCC	Forward 5′-GGCTCGATTGTTATTTCCAC -3′	60
	Reverse 5′-GGTTGTAGAATTAAGAATAGC-3′	
IL-6	Forward 5′-CCATCCAGTTGCCTTCTTGG-3′	60
	Reverse 5′-CTCCTCTCCGGACTTGTGAA-3′	
MIC-1	Forward 5′-CCCTGCAGTCCGGATACTC-3′	60
	Reverse 5′-GAACAGAGCCCGGTGAAG-3′	

Supplementary table 2. NMR data of CMSP

No.	^{1}H NMR	^{13}C NMR
1	/	126.369
2	7.47(1H,dd,*J*=2.5,9.5Hz)	117.072
3	6.78(1H, dd,*J*=2.5,9.5Hz)	132.008
4	/	162.452
5	6.78(1H, dd,*J*=2.5,9.5Hz)	132.008
6	7.47(1H,dd,*J*=2.5,9.5Hz)	117.072
1'	9.50(1H,d,*J*=8.0Hz)	196.184
2'	7.52(1H, d,*J*=15.5Hz)	126.998
3'	6.55(1H, d,*J*=8.0,16.0Hz)	156.003

Supplementary Figure. 1.

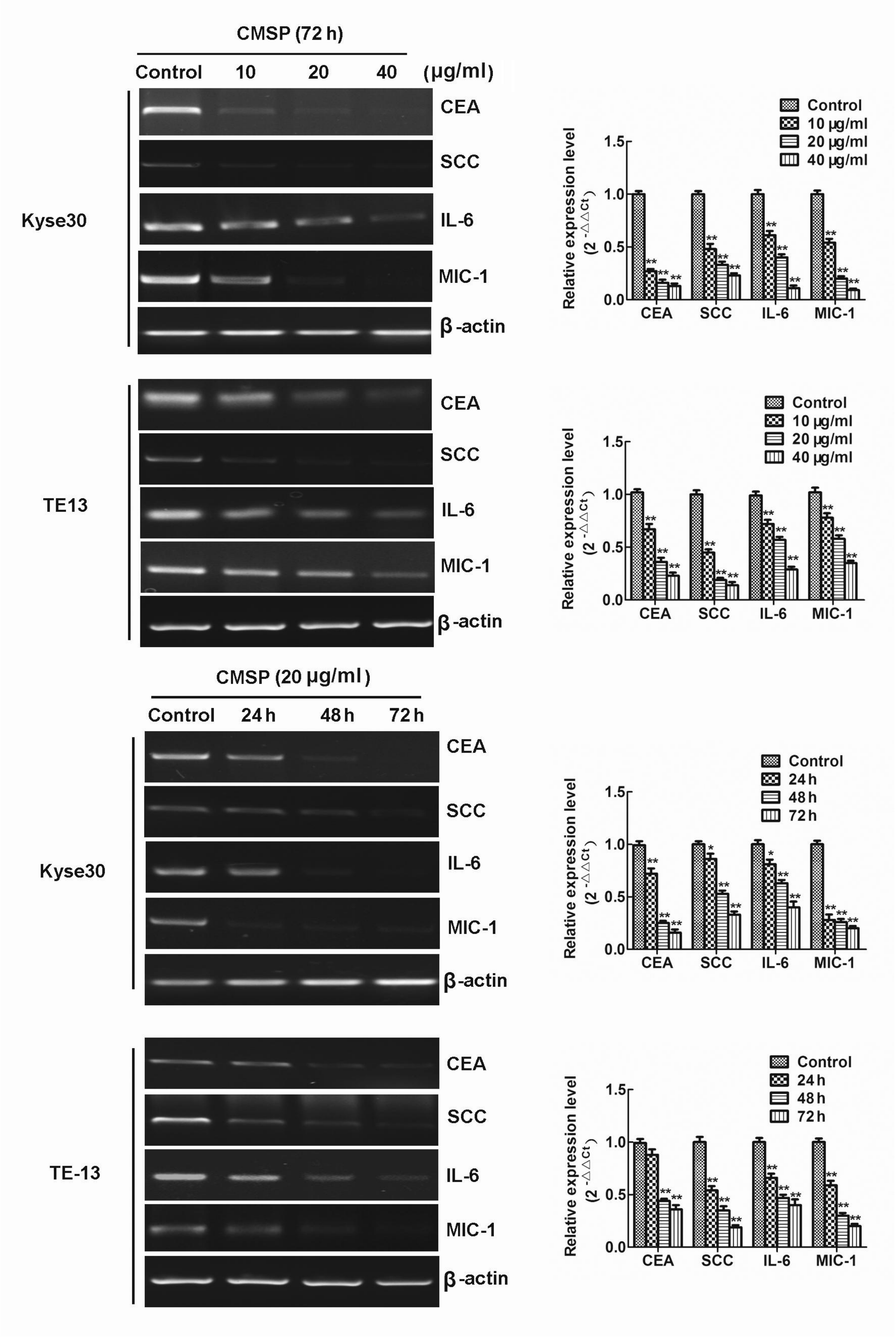

Supplementary Figure. 2.

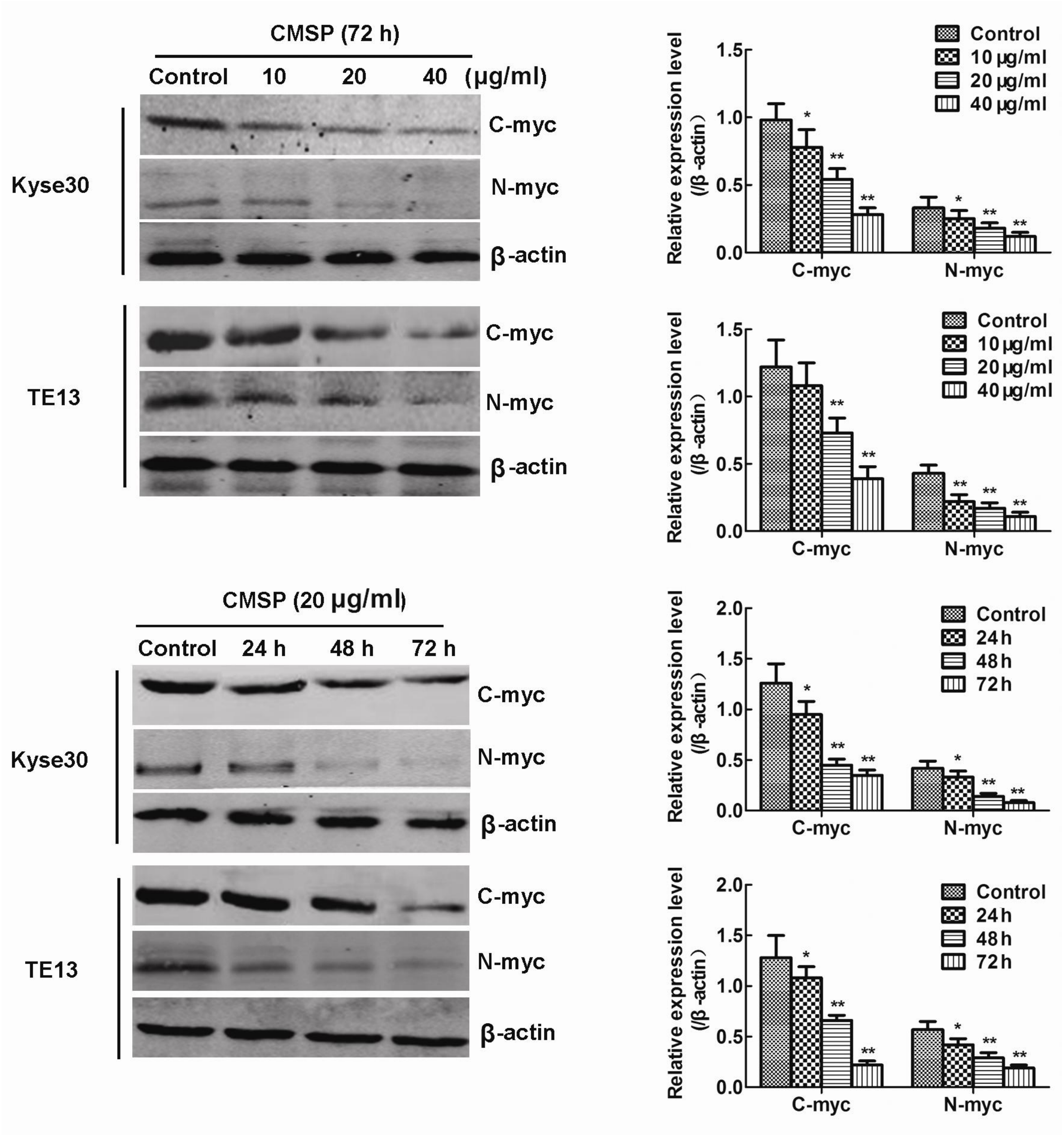

Supplementary Figure. 3.

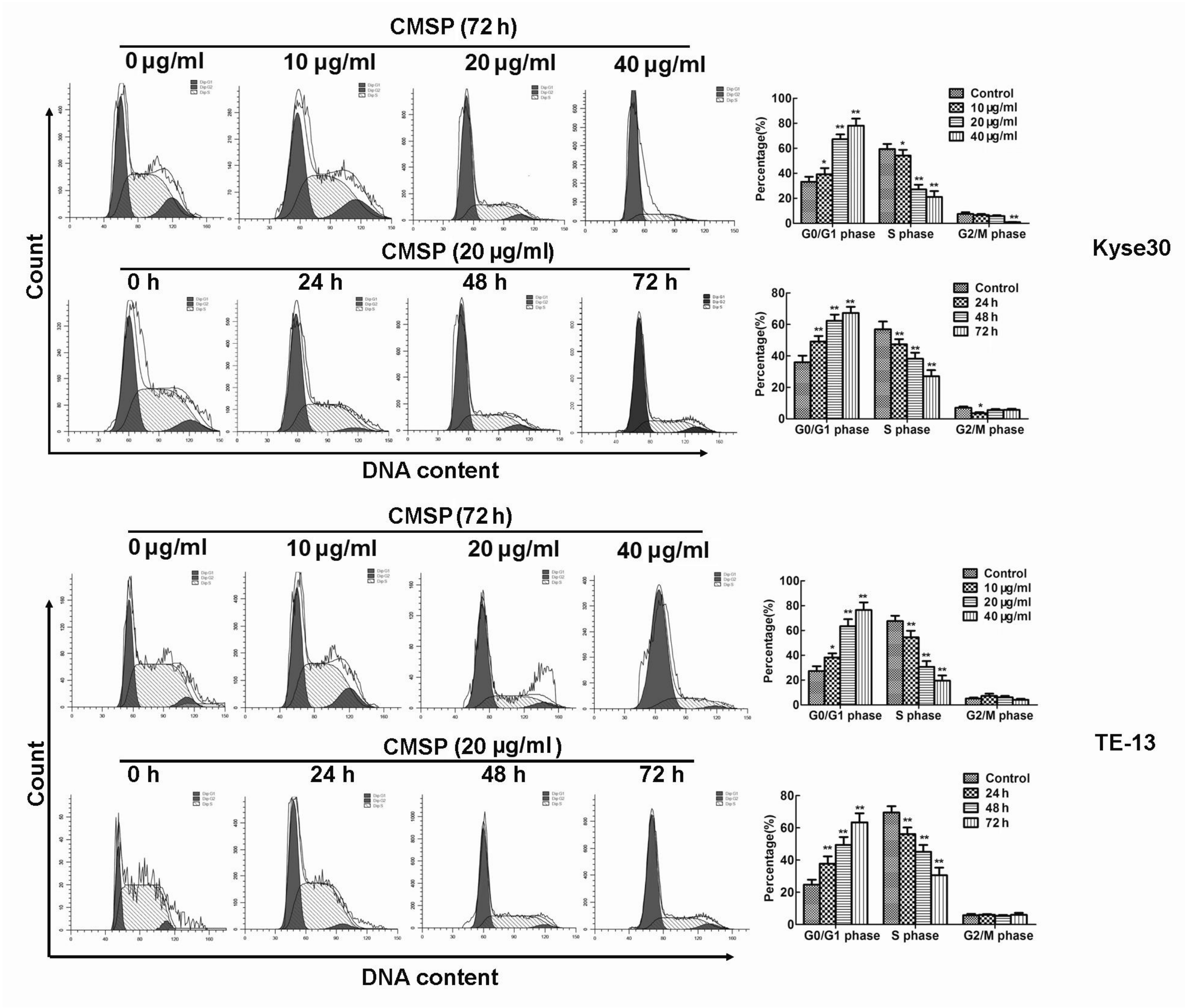

Supplementary Figure. 4.

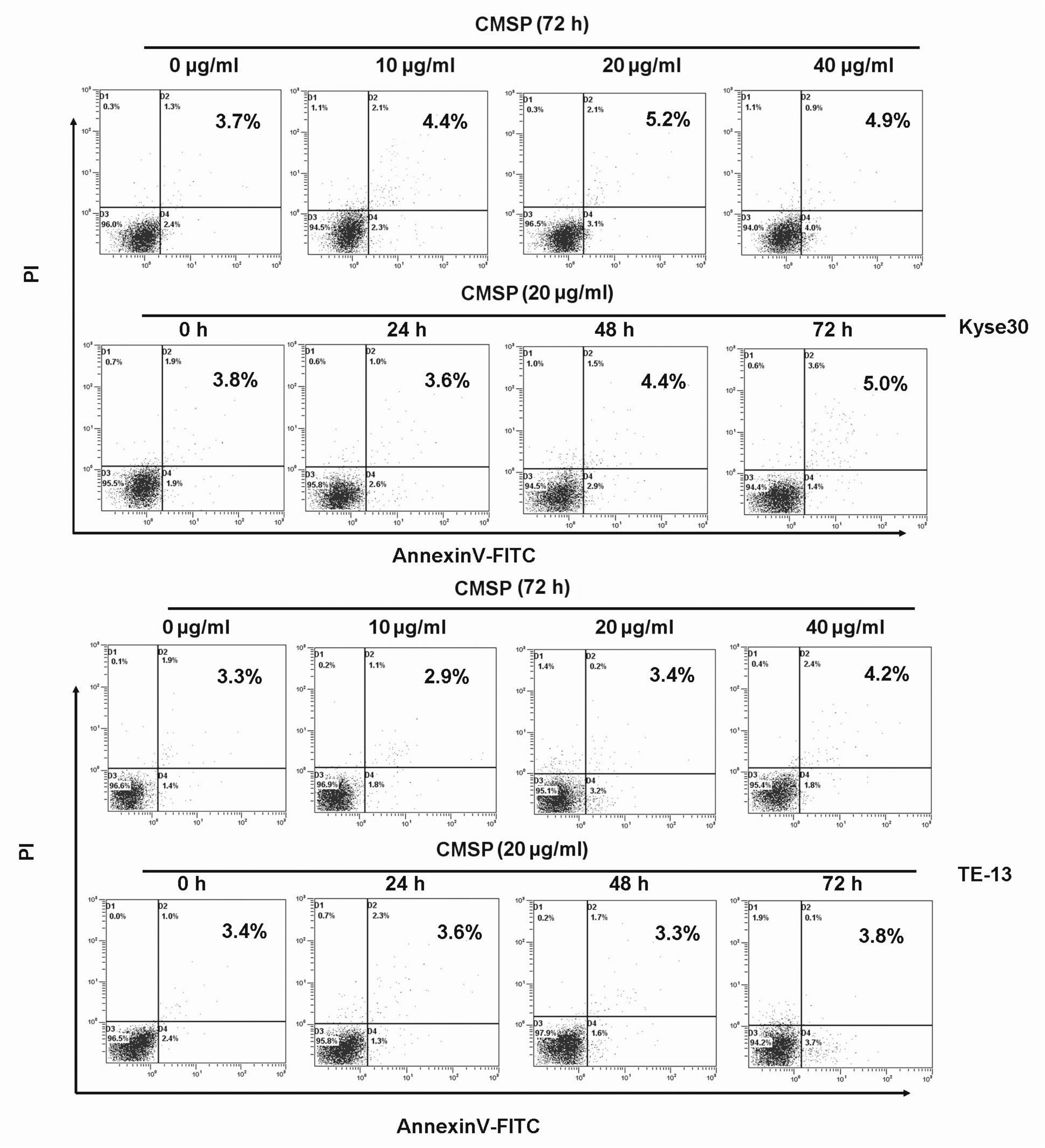

Supplementary Figure. 5.

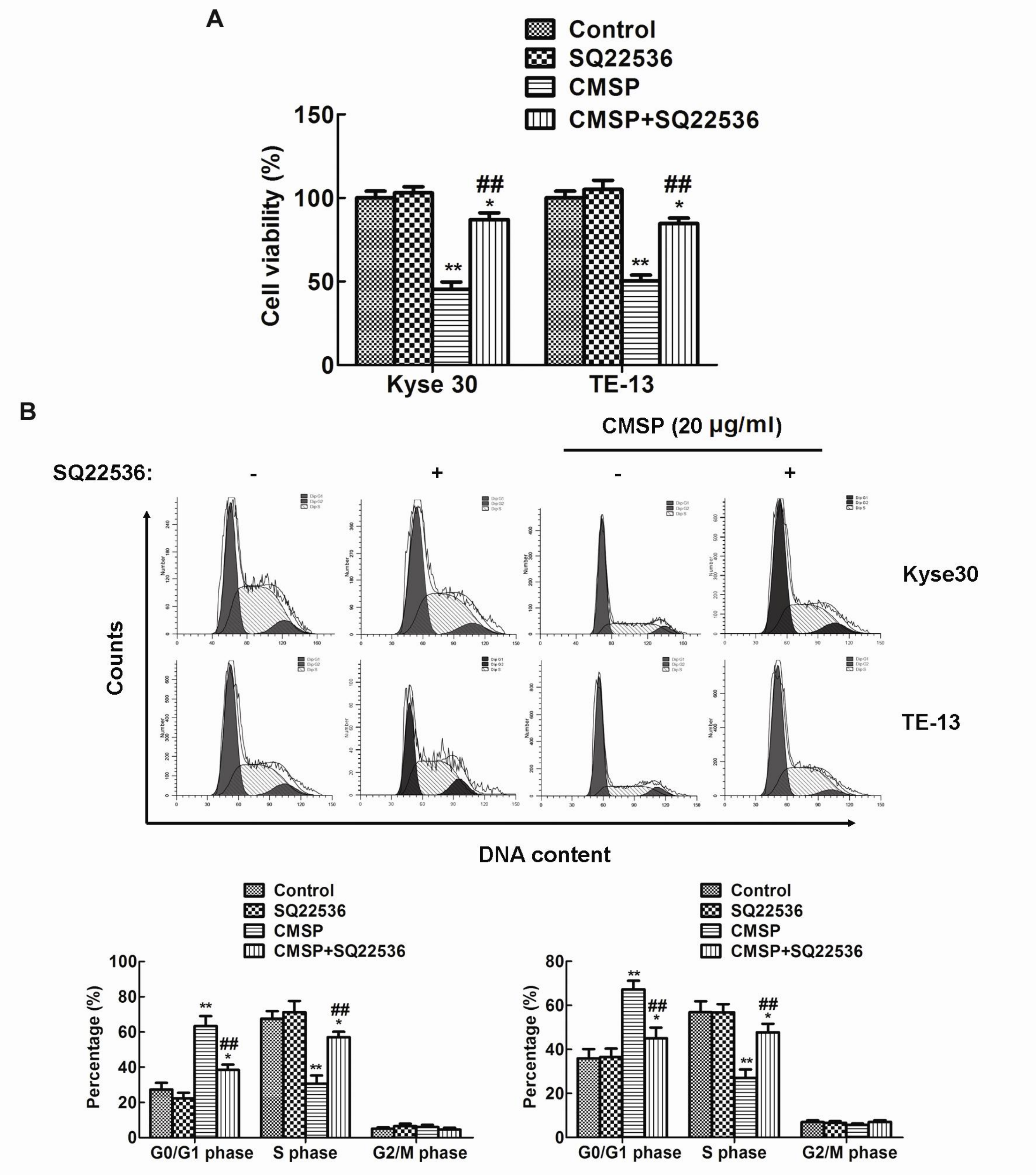

Supplementary Figure. 6.

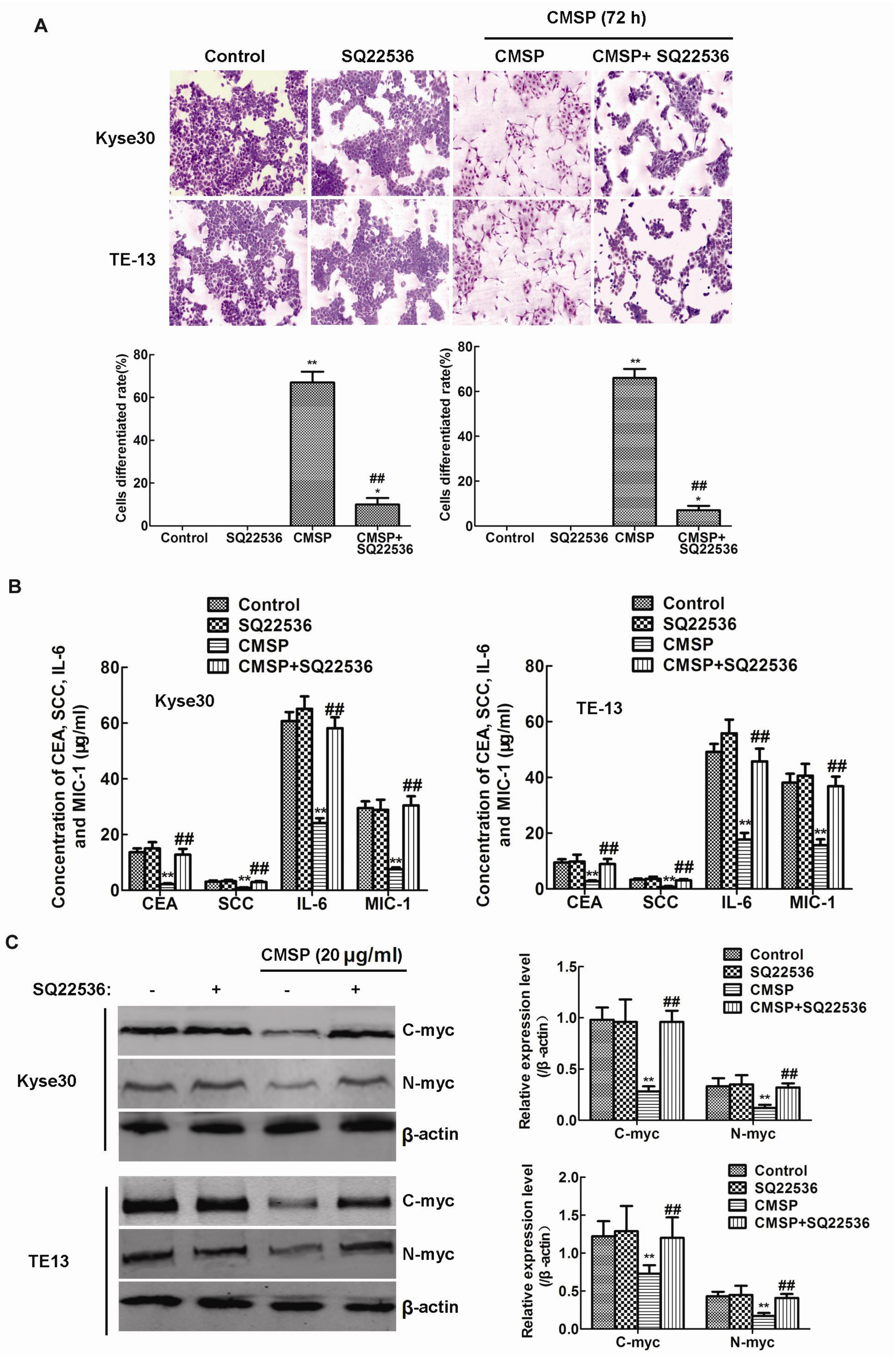
A
CMSP (72 h)
Control
SQ22536
CMSP
CMSP+ SQ22536
Kyse30
TE-13
Cells differentiated rate(%)
0
20
40
60
80
Control
SQ22536
CMSP
CMSP+ SQ22536
**
*
##
B
Control
SQ22536
CMSP
CMSP+SQ22536
Concentration of CEA, SCC, IL-6 and MIC-1 (μg/ml)
Kyse30
TE-13
CEA
SCC
IL-6
MIC-1
**
##
C
CMSP (20 μg/ml)
SQ22536: - + - +
C-myc
N-myc
β-actin
Kyse30
TE13
Relative expression level (/β-actin)
0.0
0.5
1.0
1.5
2.0
Control
SQ22536
CMSP
CMSP+SQ22536
C-myc
N-myc
**
##

Supplementary Figure. 7.

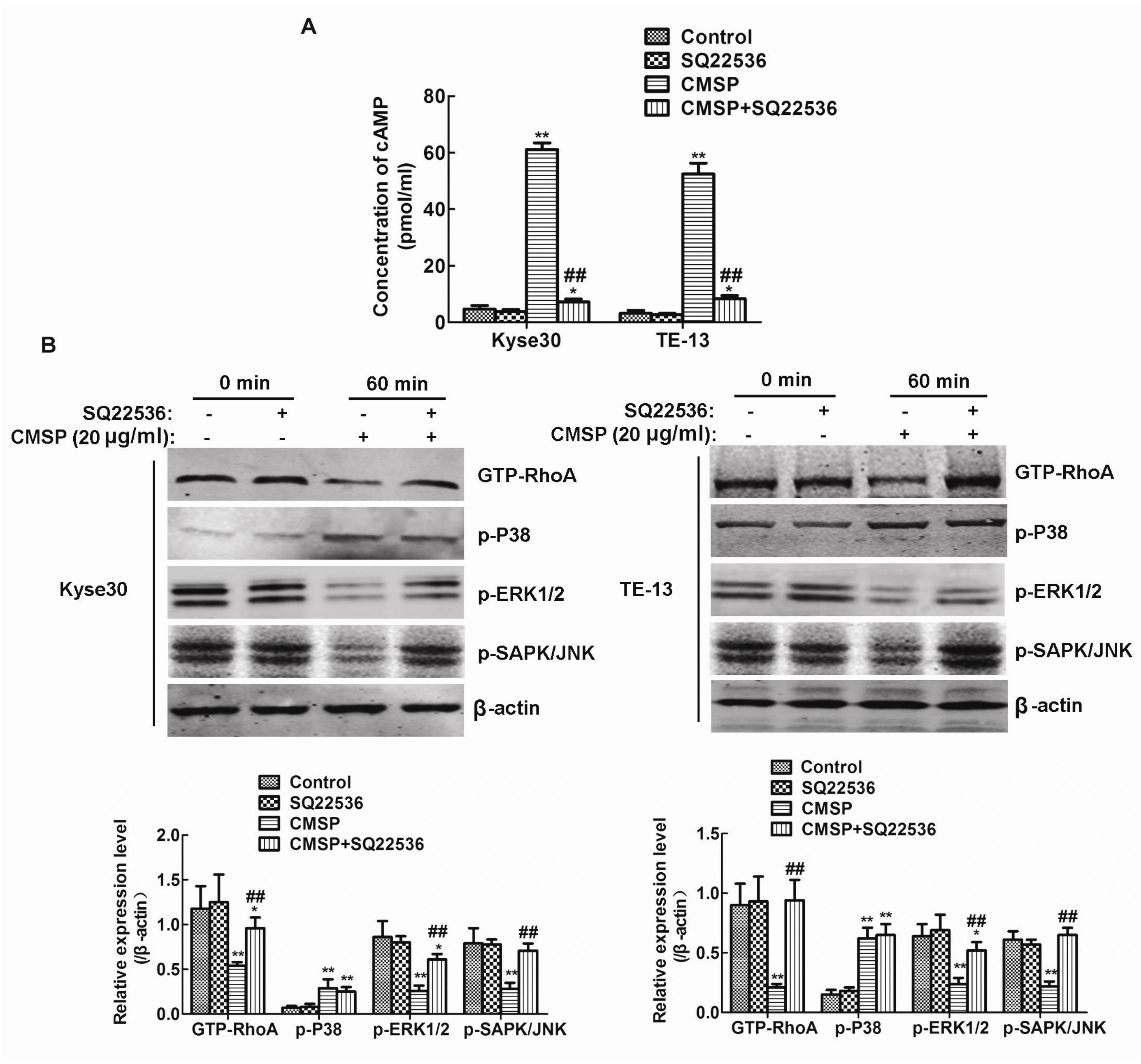

Figure legends

Supplementary Figure. 1. Effects of CMSP on tumour-related antigen and malignant marker expression of Kyse30 and TE-13 cells following CMSP treatment.

Expression of CEA, SCC IL-6 and MIC-1 mRNA in Kyse30 and TE-13 cells treated with different concentrations (10, 20 or 40 μg/ml) of CMSP was determined using RT-qPCR. β-Actin served as a loading control. The data presented are means±SD from at least three independent experiments. *P<0.05, **P<0.01, compared with the

control group.

Supplementary Figure. 2. Effects of CMSP on C-myc and N-myc expression in Kyse30 and TE-13 cells following exposure to CMSP.

The expression levels of C-myc and N-myc, two malignant markers of tumours, in Kyse30 and TE-13 cells treated with different concentrations (10, 20 or 40 μg/ml) of CMSP were determined by western blotting. β-Actin served as a loading control. The data presented are means±SD from at least three independent experiments. *P<0.05, **P<0.01, compared with the control group.

Supplementary Figure. 3. Effects of CMSP on the cell cycle distribution of Kyse30 and TE-13 cells.

Effect of CMSP on the cell cycle distribution of Kyse30 and TE-13 cells detected by FCM analysis. Representative histograms of PI-stained cells. The data presented are means±SD from at least three independent experiments. *P<0.05, **P<0.01, compared with the control group.

Supplementary Figure. 4. Effects of CMSP on the apoptosis of Kyse30 and TE-13 cells.

The effect of CMSP on the apoptotic rates of Kyse30 and TE-13 cells was detected by flow cytometry analysis.

Supplementary Figure. 5. Effects of inhibitor of cAMP on CMSP-mediated proliferation inhibition and cycle arrest of Kyse30 and TE-13 cells.

(A) Effect of SQ22536 on proliferation inhibition of Kyse30 and TE-13 cells induced by 20 μg/ml CMSP for 72 h. The data presented are means±SD from at least three independent experiments. *P<0.05, **P<0.01, compared with the control group. # P<0.05, ##P<0.01, compared with the CMSP group.

(B) Effect of SQ22536on cycle arrest of Kyse30 and TE-13 cells induced by 20 μg/ml CMSP for 72 h. The data presented are means±SD from at least three independent experiments. *P<0.05, **P<0.01, compared with the control group. ##P<0.01, compared with the CMSP group.

Supplementary Figure. 6. Effects of inhibitor of cAMP on CMSP-mediated morphology changes and differentiation of Kyse30 and TE-13 cells.

(A) Effect of inhibitor for cAMP (SQ22536) on morphology changes of Kyse30 and TE-13 cells induced by 20 μg/ml CMSP for 72 h. (×100).

(B) Effect of SQ22536 on CEA, SCC, IL-6 and MIC-1 secretion inhibition of Kyse30 and TE-13 cells mediated by 20 μg/ml CMSP for 72 h. The data presented are means±SD from at least three independent experiments. *P<0.05, **P<0.01, compared with the control group. ##P<0.01, compared with the CMSP group.

(C) Effect of SQ22536 on expression levels of C-myc and N-myc, two malignant markers of tumours, in Kyse30 and TE-13 cells treated with 20 μg/ml CMSP for 72 h. The data presented are means±SD from at least three independent experiments.

*P<0.05, **P<0.01, compared with the control group. ##P<0.01, compared with the CMSP group.

Supplementary Figure. 7. Effects of SQ22536 on GTP-RhoA expression and the phosphorylation of MAPKs in Kyse30 and TE-13 cells treated with CMSP.
(A) Effect of SQ22536 on cAMP secretion of Kyse30 and TE-13 cells treated with 20 μg/ml CMSP for 60 min. The data presented are means±SD from at least three independent experiments. *P<0.05, **P<0.01, compared with the control group. ## P<0.01, compared with the CMSP group.
(B) The effect of SQ22536 on expression changes of GTP-RhoA, phosphorylated ERK, p38, and JNK kinase proteins mediated by CMSP were assayed by western blotting. β-Actin was used as an internal control. The data presented are means±SD from at least three independent experiments. *P<0.05, **P<0.01, compared with the control group. ##P<0.01, compared with the CMSP group.
